# Great Tits (*Parus major*) Reduce Caterpillar Damage in Commercial Apple Orchards

**DOI:** 10.1371/journal.pone.0000202

**Published:** 2007-02-07

**Authors:** Christel M.M. Mols, Marcel E. Visser

**Affiliations:** Netherlands Institute of Ecology (NIOO-KNAW), Heteren, The Netherlands; University of Edinburgh, United Kingdom

## Abstract

Alternative ways to control caterpillar pests and reduce the use of pesticides in apple orchards are in the interest of the environment, farmers and the public. Great tits have already been shown to reduce damage under high caterpillar density when breeding in nest boxes in an experimental apple orchard. We tested whether this reduction also occurs under practical conditions of Integrated Pest Management (IPM), as well as Organic Farming (OF), by setting up an area with nest boxes while leaving a comparable area as a control within 12 commercial orchards. We showed that in IPM orchards, but not in OF orchards, in the areas with breeding great tits, apples had 50% of the caterpillar damage of the control areas. Offering nest boxes to attract insectivorous passerines in orchards can thus lead to more limited pesticide use, thereby adding to the natural biological diversity in an agricultural landscape, while also being economically profitable to the fruit growers.

## Introduction

Biological control of pests is becoming increasingly important as an answer to the resistance of harmful insects to pesticides, the adverse public attitudes to pesticides and the increasing restriction of their use due to legislation [1]. The main focus in the search for biological pest control agents has been on parasitoid insects [2] and predatory arthropods such as predatory mites, earwigs, lacewings, mirids and anthocorids [1]. The potential contribution of vertebrate predators such as birds has generally been overlooked, mainly because of their presumed lack of a sufficient numerical response to outbreaks of pests. However, most of the studies on birds as biological pest control agents do show a reduction in the population size of harmful insect species [review see 3,4,5]. These studies therefore show that birds have the potential as biological pest control agents, particularly in crops such as apples.

The great tit *Parus major* L. is a common species in the Netherlands and breeds readily in nest boxes. Putting up nest boxes can easily enhance the local density of great tits [6 and personal data]. Within the breeding season great tits mainly forage for caterpillars, which is the preferred food for their nestlings [7]–[10]. Caterpillars in orchards, such as winter moths *Operophtera brumata* L. and tortricid moths, are key pests [1], [2]. These features together suggest that great tits can serve as biological control agents for caterpillars in orchards. Mols and Visser [4] showed that foraging by great tits reduced caterpillar damage on apples in an experimental orchard with high caterpillar densities. The question remains, however, whether great tits can also be effective in reducing damage under commercial management with lower caterpillar densities.

In this study we investigated whether great tits can reduce caterpillar damage in commercially managed apple orchards, with either Integrated Pest Management (IPM) or Organic Farming (OF), by measuring damage levels of the apples in areas with and without breeding great tits.

## Results

**Figure 1:**
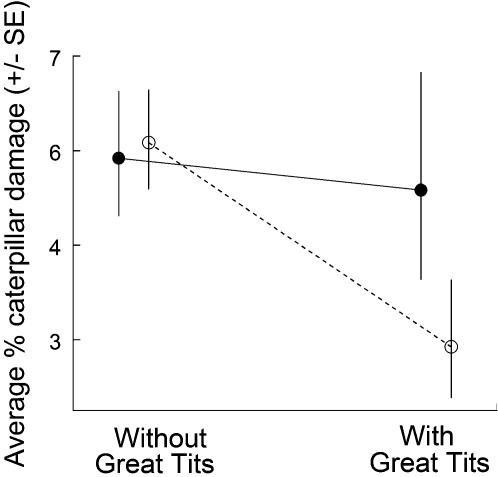
Caterpillar damage to apples (percentage of damaged apples) in 2-ha plots within 12 apple orchards (in total 19 orchard-years) with and without breeding great tits (Parus major). Solid lines and closed points are for the 6 Organic Farming (OF) orchards, broken lines and open points are for the 6 Integrated Pest Management (IPM) orchards.

There was a significant effect of the presence versus absence of great tits: a significantly lower percentage of apples was damaged in the area with nest boxes compared to the control area in the same orchard (Table S1; repeated measures ANOVA with binominal errors: treatment: χ^2^(1) = 4.71, P = 0.03, orchard type x treatment χ^2^(1) = 3.88, P = 0.049; test without interaction: treatment: χ^2^(1) = 4.03, P = 0.045). There was no damage reduction in the OF orchards but a very substantial reduction in the IPM orchards, where the damage in the areas with breeding great tits was 50% of the damage in the control areas (5.8% *vs* 2.9%; Fig. 1).

The effect of the number of breeding pairs of great tits on damage reduction could not be fitted when treatment also was included in the model as this number was always 0 in the control areas. Tested in the absence of treatment, breeding density had a significant effect on the amount of caterpillar damage to the apples (χ^2^(1) = 4.18, P = 0.041; interaction with type of orchard marginally non-significant χ^2^(1) = 3.71, P = 0.054). The model with treatment had a higher log likelihood than the model with number of breeding pairs and was thus preferred.

## Discussion

Although some studies suggest that birds do not remove prey that occur at low densities [11]–[13], our study shows that great tits are able to reduce damage at low caterpillar densities in IPM orchards (Fig. 1). Surprisingly, we were unable to show a damage reduction in the OF orchards, although the caterpillar density was higher in the OF orchards (χ^2^(1) = 7.27, P = 0.007). We found earlier that in an experimental orchard, with even higher caterpillar densities, there again was a reduction in caterpillar damage when Great Tits were allowed to forage in the apple trees (Mols & Visser, 2002).

Fruit growers with IPM orchards thus benefit from the presence of great tits in their orchards even without changing their normal management. Reducing caterpillar damage in the orchard by offering nest boxes to great tits is an extremely low cost measure (€20 per ha per year – 10 nest boxes a €20 per ha, which need to be replaced every 10 years) that can reduce damage by up to 50 %, i.e. an increase in yield of undamaged apples of 1200 kg per ha (average production is 40000 kg per ha per year).

Additionally, our findings imply that the threshold caterpillar densities at which pesticides need to be applied can be raised when great tits are breeding in orchards. For example, when great tits are absent from an orchard a damage level of 3.5% is reached with an initial density of four caterpillars per tree, while when two to four pairs of great tits are foraging in an orchard the same damage occurs with eight caterpillars per tree. Offering nest boxes to attract insectivorous passerines in orchards can thus lead to more limited pesticide use, thereby adding to the natural biological diversity in an agricultural landscape, while also being economically profitable to the fruit growers.

## Material and Methods

We measured caterpillar densities at the start of the growing season and damage levels to apples at harvest in two similar areas of 2 ha each, one of which was randomly assigned to the nest box treatment, in 12 Dutch apple orchards over a period of four years (1997–2000). Plots were at least 500 m apart, too far for great tits breeding in the nest box plot to forage in the control plot. In Dutch apple orchards there are two management regimes, and we used orchards under both types of regimes. We used six orchards with Integrated Pest Management (IPM) orchards where pesticides are used only if caterpillar densities exceed a control threshold [14]. This way large-scale caterpillar damage, which leads to considerable economic costs, is avoided in years with high caterpillar densities while in low-density years pesticide use is avoided. Furthermore, the pesticides used are mostly species-specific to avoid elimination of useful predatory mites and, if possible, other natural enemies. The other six were Organic Farming (OF) orchards where only plant-derived pesticides, natural enemies (including bacteria and viruses) and disruption of mating by pheromones are used and no synthetic pesticides, herbicides or chemical fertilisers are applied. The main apple varieties in the orchards were Elstar and Jonagold.

Nest boxes (15 to 25) were systematically placed in the inner 1.5 ha of the nest box area to attract great tits. Six orchards were sampled in one year, five in two years and one in three years (n = 19). All nest box areas had at least one pair of breeding great tits. The laying date, clutch size and number of great tit fledglings were determined by checking nest boxes weekly from mid April until all broods had fledged.

Caterpillar densities were determined on 40 trees in each control and nest box area at the end of apple bloom by counting caterpillars on the third lowest branch of each tree. Initial densities of caterpillars did not differ between the areas with nest boxes and the control areas (repeated measures ANOVA: χ^2^(1) = 0.55, P = 0.36, density higher in OF than IPM orchards χ^2^(1) = 7.27, P = 0.007).

In autumn we checked 25 apples from each of 40 trees in each of the nest box and control area (thus 2000 apples per orchard) for caterpillar spring damage, as described by De Reede et al. [15]. Caterpillar spring damage is easily distinguished from other caterpillar damage by the diagnostic corked scar tissue. The percentage of damage was calculated per tree and averaged per area (nest box and control) per orchard.

We used a repeated measure ANOVA (to maintain the link between the area with and without great tits within an orchard) with binominal errors with caterpillar damage (number of damaged apples over number of apples scored per area) as the response variable and treatment, management type (IPM or OF) and their interaction as explanatory variables. Initial caterpillar densities (average number of caterpillars per tree) were analysed with a repeated measures ANOVA with treatment, management type (IPM or OF) and their interaction as explanatory variables. In both analyses the GenMod procedure of SAS V8 was used.

## Supporting Information

Table S1 Data on caterpillar damage on apples in six Integrated Pest Management (IPM) and six Organic Farming (OF) orchards: name of the orchard, year of sampling, type of management (IPM/OF), number of breeding pairs of Great Tits, number of caterpillars per tree, treatment (control/nest box area), number of apples sampled and number of damaged apples.
(0.10 MB DOC)
