# Supplementary material for: Great Tits (Parus major) Reduce Caterpillar Damage in Commercial Apple Orchards
Source: PLoS One. 2007 Feb 7;2(2):e202. doi: 10.1371/journal.pone.0000202 (PMC1784073; doi:10.1371/journal.pone.0000202)
Supplement: Table S1 — Data on caterpillar damage on apples in six Integrated Pest Management (IPM) and six Organic Farming (OF) orchards: name of the orchard, year of sampling, type of management (IPM/OF), number of breeding pairs of Great Tits, number of caterpillars per tree, treatment (control/nest box area), number of apples sampled and number of damaged apples. (0.10 MB DOC) [file pone.0000202.s001.doc]

| Orchard | Year | Type | NoPairs | DensCater | Treatment | NoApples | NoDamaged |
| --- | --- | --- | --- | --- | --- | --- | --- |
|  |  |  |  |  |  |  |  |
| Albers | 1999 | OF | 2 | 6.42 | Nb | 1000 | 40 |
| Albers | 1999 | OF | 0 | 7.24 | Ct | 1000 | 51 |
| Albers | 2000 | OF | 2 | 8.24 | Nb | 1000 | 48 |
| Albers | 2000 | OF | 0 | 7.70 | Ct | 1000 | 32 |
| Bosch | 1999 | IPM | 5 | 5.96 | Nb | 1000 | 22 |
| Bosch | 1999 | IPM | 0 | 5.87 | Ct | 1000 | 20 |
| Erp | 1999 | IPM | 2 | 5.96 | Nb | 1000 | 17 |
| Erp | 1999 | IPM | 0 | 5.87 | Ct | 1000 | 98 |
| Flikweert | 1997 | OF | 1 | 7.39 | Nb | 1000 | 9 |
| Flikweert | 1997 | OF | 0 | 7.48 | Ct | 1000 | 9 |
| Flikweert | 2000 | OF | 4 | 6.14 | Nb | 1001 | 42 |
| Flikweert | 2000 | OF | 0 | 6.42 | Ct | 1000 | 72 |
| Groen | 1999 | IPM | 4 | 5.96 | Nb | 1000 | 7 |
| Groen | 1999 | IPM | 0 | 5.87 | Ct | 1000 | 16 |
| Janssen | 1997 | IPM | 2 | 5.94 | Nb | 1000 | 62 |
| Janssen | 1997 | IPM | 0 | 5.87 | Ct | 1000 | 141 |
| Janssen | 1999 | IPM | 3 | 5.96 | Nb | 1000 | 22 |
| Janssen | 1999 | IPM | 0 | 5.96 | Ct | 1000 | 39 |
| Luttervel | 1997 | IPM | 4 | 6.80 | Nb | 1000 | 62 |
| Luttervel | 1997 | IPM | 0 | 6.16 | Ct | 1000 | 64 |
| Peters | 1998 | OF | 3 | 7.40 | Nb | 1000 | 70 |
| Peters | 1998 | OF | 0 | 7.33 | Ct | 1000 | 80 |
| Peters | 1999 | OF | 4 | 6.24 | Nb | 888 | 77 |
| Peters | 1999 | OF | 0 | 6.05 | Ct | 1001 | 84 |
| Peters | 2000 | OF | 4 | 7.70 | Nb | 1000 | 59 |
| Peters | 2000 | OF | 0 | 8.24 | Ct | 999 | 76 |
| Poley | 1999 | OF | 4 | 6.42 | Nb | 1000 | 48 |
| Poley | 1999 | OF | 0 | 6.69 | Ct | 1000 | 33 |
| Poley | 2000 | OF | 5 | 6.24 | Nb | 1000 | 98 |
| Poley | 2000 | OF | 0 | 6.51 | Ct | 1003 | 139 |
| Stoker | 1999 | OF | 4 | 6.69 | Nb | 1000 | 24 |
| Stoker | 1999 | OF | 0 | 6.42 | Ct | 1000 | 25 |
| Stoker | 2000 | OF | 6 | 6.33 | Nb | 1000 | 43 |
| Stoker | 2000 | OF | 0 | 7.06 | Ct | 1000 | 36 |
| VdRijdt | 1997 | IPM | 4 | 6.67 | Nb | 950 | 10 |
| VdRijdt | 1997 | IPM | 0 | 6.60 | Ct | 1000 | 27 |
| Korstanje | 1997 | OF | 3 | 11.05 | Nb | 1237 | 78 |
| Korstanje | 1997 | OF | 0 | 6.82 | Ct | 1961 | 104 |
